# Dynamic splinting in wrist extension following distal radius fractures

**DOI:** 10.1186/1749-799X-5-53

**Published:** 2010-08-06

**Authors:** Stacey H Berner, F Buck Willis

**Affiliations:** 1 Advanced Centers for Orthopaedic Surgery & Sports Medicine, 10 Crossroads #210 Owings Mills, MD 21117, USA; 2 University of Phoenix: Axia College; Health Sciences, Adjunct Instructor and Dynasplint Systems, Inc, Clinical Research, PO Box 1735 San Marcos TX 78667, USA

## Abstract

**Background:**

Wrist flexion contracture is a common pathology which presents secondary to distal radius fractures. Joint stability, restoration and early mobilization are frequently achieved through surgical treatment after such an injury. The purpose of this retrospective study was to evaluate the initial effect of dynamic splinting on wrist extension (active range of motion), in both surgical and non-surgical patients following distal radius fractures.

**Methods:**

Records were obtained from 133 patients who were treated with a Wrist Extension Dynasplint (WED) following distal radius fractures, between May 2007 and May 2009. Forty-two of these patients received surgical treatment for their fractures. This study specifically examined the initial usage of the WED as a home therapy. The retrospective analysis included categorization of patients who received the WED exclusively vs. patients who received WED treatment with concurrent hand therapy; surgical categorization included surgical patients vs. nonsurgical patients.

**Results:**

There was a significant improvement in maximal active range of motion (AROM) for all patients (P < 0.0001) after a mean duration of 3.9 weeks of dynamic splinting. Patients showed a mean 62% increase in active extension. There was not a significant difference between patients who had received surgical treatment for the fracture vs. nonsurgical.

**Conclusion:**

This dynamic splinting modality contributed 138 to 185 hours of stretching at the end range of motion for these patients in their first month following fracture. This unique regime is considered directly responsible for significant gains in AROM.

## Introduction

Contracture is seen commonly following Distal Radius Fractures (DRF), and surgical management is being used on an increasing number of DRF patients [1,2]. Greater frequency of surgical procedures may come from the desire to help patients restore stability, correct articular malalignment, and regain mobility more expediently [3-7].

Patient satisfaction through increased joint mobility has been the primary outcome measure in numerous studies that examined the current therapeutic protocols for treating DRF [8-13] and protocols supplemented with prolonged durations of therapeutic stretching [14-19]. Franco et al conducted a randomized, cross-over, cohort study of 45 asymptomatic patients treated with static splints (which restricts mobility similar to immobilization) [4]. After treatment in a restrictive, static splint, Franco et al concluded that immobilization creates incrementally significant functional limitations.

Prolonged stretching has been shown to have a significant effect on connective tissue in molecular examination [17] and clinical trials [18,19]. Dynamic splinting employs passive, prolonged stretching which has proven responsible for contracture reduction in other joints and pathologies [13-16]. The purpose of this retrospective study was to evaluate the initial effect of dynamic splinting on wrist extension (active range of motion), in both surgical and non-surgical patients following distal radius fractures.

## Materials and methods

### Patients

This retrospective study examined the records of 133 DRF patients (78 women, 55 men; mean age 53 ± 17.6) who were treated with dynamic splinting for contracture reduction following distal radius fractures. Forty-two of these patients received this treatment following surgical management of DRF. All patients treated with this modality were fit within one week of the diagnosis of wrist flexion contracture, secondary to DRF. Patients were prescribed nightly use of the dynamic splinting modality. (All patients gave informed consent for use of their records in this retrospective study.) See demographics in Table 1.

**Table 1:** Demographics

Parameter	n
Total Patients	133

Surgical Patients (%)	42 (31.6%)

Age (yrs)	53 ± 17.6

Female (%)	78 (58.6%)

Pts who had Physical Therapy (%)	54 (40.6%)

White (%)	88%

Other (%)	6%

Black (%)	2%

Hispanic (%)	4%

### Intervention

**Figure 1:**
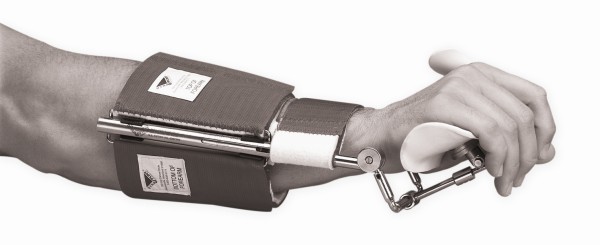
**Wrist Extension Dynasplint**.

Patients' initial introduction to the Wrist Extension Dynasplint (WED) system, [Dynasplint Systems, Inc., Severna Park, MD, USA] included customized fitting (wrist length, width, and girth so that the force and counter force straps could be properly aligned) and training on donning and doffing of the device. (See figure 1.) Verbal and written instructions were provided throughout the duration of treatment for safety, general wear and care, and tension setting goals based on patient tolerance.

Each patient initially wore the WED for 4-6 continuous hours at an initial tension setting of #2 (0.1 foot pounds of torque). This duration was for acclimatization to the system; then patients were instructed to wear the WED system at night while sleeping for 6-8 hours of continuous wear. After each patient was comfortable wearing the unit for one week at tension level #2, they were instructed to increase the tension level to #3 (0.3 ft lbs.) and make continual increases every two weeks. If prolonged soreness followed a session (soreness for more than 15 minutes) the patient was instructed to decrease the tension one half a setting for two days until they were comfortable wearing it for 6-8 hours at the new tension setting. The majority of all patients reached level #5 (0.8 foot pounds of torque) by the end of two months. All range of motion measurements were recorded by the prescribing clinician.

### Statistical Methods

The dependent variable in this study was the change in wrist extension AROM and the independent variables were the patient treatment categories, post surgical vs. non surgical and concurrent physical therapy plus WED vs. exclusive WED treatment. Statistical data analysis was accomplished using a repeated measures analysis of variance (ANOVA) with Post-hoc T-tests. Data analysis was done independently by Dr. Ram Shanmugam, a biostatistics professor at Texas State University, San Marcos, TX.

## Results

**Figure 2:**
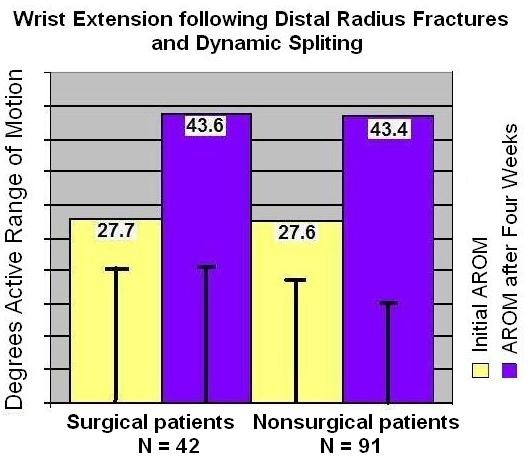
**Results**.

There was a significant improvement in motion for all patients (P = 0.0001, T = 10.126, df 132; see figure 2). The mean increase was 16° AROM for all patients (SD 2.1). A normal statistical distribution of data was seen in both the initial AROM measurements and the final AROM, and these measurements were highly correlated. There was not a significant difference between patients who received previous surgical treatment or Physical Therapy (PT) plus WED, (74% PT+WED, N = 101, P > 0.05). The results showed no significant difference among gender or in the duration between fracture and WED treatment (Mean 3.9 weeks, Range 3-20 weeks, SD = 3.87, P > 0.05).

## Discussion

This modality was responsible for regaining 62% increased active range of motion which will directly affect function. The efficacy of dynamic splinting for contracture reduction was similar to results from the Carpal Tunnel study by Berner and Willis. The results of that study showed efficacy of dynamic splinting in reducing symptoms from carpal tunnel syndrome [15]. The outcome measured changes in Levine-Katz function/pain survey and nerve conduction, which showed statistically significant differences [15] The benefits of WED in this retrospective cohort study were comparable to results seen in a case report on dynamic splinting for contracture following radial nerve injury [13]. McKee and Nguyen's case described a 76 year old patient who suffered a radial nerve injury following a shoulder replacement. They found that dynamic splinting helped this patient regain motor functions that were previously disabled by the excessive wrist flexion contracture [13]. Molecular analysis has proven prolonged stretching responsible for connective tissue elongation [14-16], as employed in dynamic splinting.

The lack of difference between surgical patients vs. non surgical patients supports the hypothesis that contracture was caused by the combined effect of the distal radius fracture and the associated soft tissue injury. Several manuscripts have recommended studies examine increases in function following treatment for a distal radius fracture [1-3]. The duration of WED treatment ranged from 3 to 20 weeks and the mean duration may be skewed because of patients who discontinued using the WED in less than two months, after full ROM was restored.

The physical therapists were not blinded so their methods included the WED as the primary stretching protocol; therefore the therapists spent more time on higher therapeutic protocols for weight bearing, load bearing, and dexterity. Time saved in manual stretching due to the assistance of the dynamic splint was often dedicated to motor dexterity training for handwriting. Since there was not a significant difference with physical therapy, each surgeon should examine the specific needs. In this study the Medicare co-payment for each patient included $20/mo for the WED vs. $200 to $500/mo in co-payments for PT 3/wk. That savings would be substantial for the patients and insurance providers.

The limitations of this study include lack of a control arm. While duration from fracture to contracture was not categorized, all patients were fit with WED within one week from diagnosis of wrist flexion contracture, secondary to DRF. Clinicians often discredit case series manuscripts because such studies lack control group(s) and can have selection bias. However, this study did examine different cohort treatment groups (surgical vs. nonsurgical and physical therapy with dynamic splinting vs. exclusive dynamic splinting) [20]. This study was independent of "measurement bias" because all measurements used in data analysis were taken by clinicians, before initiation of this retrospective investigation.

Kooistra et al wrote a detailed description of the strengths and weaknesses of Case Series studies where they described that such studies can effectively generate a hypothesis for future controlled trials and prove safety of a new protocol [20]. They also described that a retrospective design more clearly reflects what is seen in routine clinical practice because treatment selection was chosen by the surgeon and patient, not by a randomized table. This fact is the cornerstone to their proclamation that retrospective cohort series studies have high external validity [20].

This retrospective study on WED for contracture reduction, secondary to DRF showed safety as there was only 1 in 133 incidence of skin breakdown and no report of significant adverse events from these treatments. This study was the first investigation of dynamic splinting for contracture reduction following distal radius fractures, and the authors recommend this work be followed by a randomized, controlled trial to measure empirical efficacy of this modality in contracture reduction.

## Competing interests

There was no extramural funding for this study. SHB had no conflict or competing interest, and has not received any funds or compensation for his participation in this study. FBW is employed by the parent company of Dynasplint Systems but he has no stock, options, or ownership in either company.

## Authors' contributions

All authors contributed equally to this work. SHB and FBW both participated in the design

of the study and manuscript. FBW was responsible for the literature review and manuscript development. The data analysis was done independently by a biostatistical professor for Texas State University, Dr Ram Shanmugam. All authors read and approved the final manuscript.
